# Typification of *Oxalisbowiei* W.T.Aiton ex G.Don (Oxalidaceae)

**DOI:** 10.3897/phytokeys.119.33280

**Published:** 2019-03-15

**Authors:** Quentin Groom

**Affiliations:** 1 Meise Botanic Garden, Nieuwelaan 38, 1860 Meise, Belgium Meise Botanic Garden Meise Belgium

**Keywords:** *Oxalis bowii*, *
Oxalis bowieana
*, Bowie’s woodsorrel, nomenclature, lectotype

## Abstract

*Oxalisbowiei* W.T.Aiton ex G.Don (Oxalidaceae) from South Africa was described in 1831, but has not been typified. Although no preserved material was mentioned in the original description, an illustration by Thomas Duncanson painted a few years earlier would have been available to W.T. Aiton at the time he described it and it matches his description. Therefore this illustration is designated as the lectotype for *Oxalisbowiei*.

## Introduction

*Oxalisbowiei* W.T.Aiton ex G.Don (Oxalidaceae), commonly known as Bowie’s woodsorrel, is a bulbous perennial from KwaZulu-Natal and Eastern Cape Province of South Africa. It is an attractive flowering plant and is occasionally grown horticulturally in mild temperate climates (Brickell and Aspden 1994). It has also become naturalised in Australia, Europe, North America and Japan (Carnevali et al. 2018; Conn et al. 1999; Nesom 2017; Thevenot et al. 2018; Yokohata 2002). Indeed, this paper is derived from other research we are conducting on the biology of invasive *Oxalis* in Europe.

**Figure 1.**
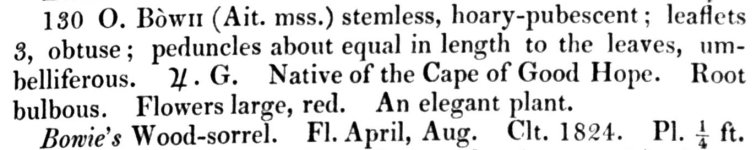
The original description of *Oxalisbowiei* W.T.Aiton ex G.Don from A general history of the dichlamydeous plants by George Don. Taken from the Biodiversity Heritage Library https://biodiversitylibrary.org/page/390566 (Don 1831).

*Oxalisbowiei* was collected by, and named after, James Bowie (circa 1789–2 July 1869) (Loddiges 1832). Bowie was employed by the Royal Botanic Gardens, Kew and was sent as a botanical collector to the Cape of Good Hope between 1817 and 1823 (Smith and van Wyk 1989). *Oxalisbowiei* was formally named in a publication by George Don (1798–1856) and attributed to W.T.Aiton, who gave the epithet *bowii* (Don 1831) (Fig. 1). The description of George Don refers to a manuscript by Aiton as “Ait. mss.”. We know this refers to William Townsend Aiton (1766–1849), rather than his father William Aiton (1731–1793), because the latter would not have not known James Bowie. Bowie was only an infant when William Aiton Sr. died.

Although the original published epithet was *bowii*, this can be legitimately corrected to *bowiei* to form a substantival epithet, *bowiei*, following articles 60.1 and 60.8 of the International Code of Nomenclature for algae, fungi, and plants (ICN) (Turland et al. 2018).

**Figure 2.**
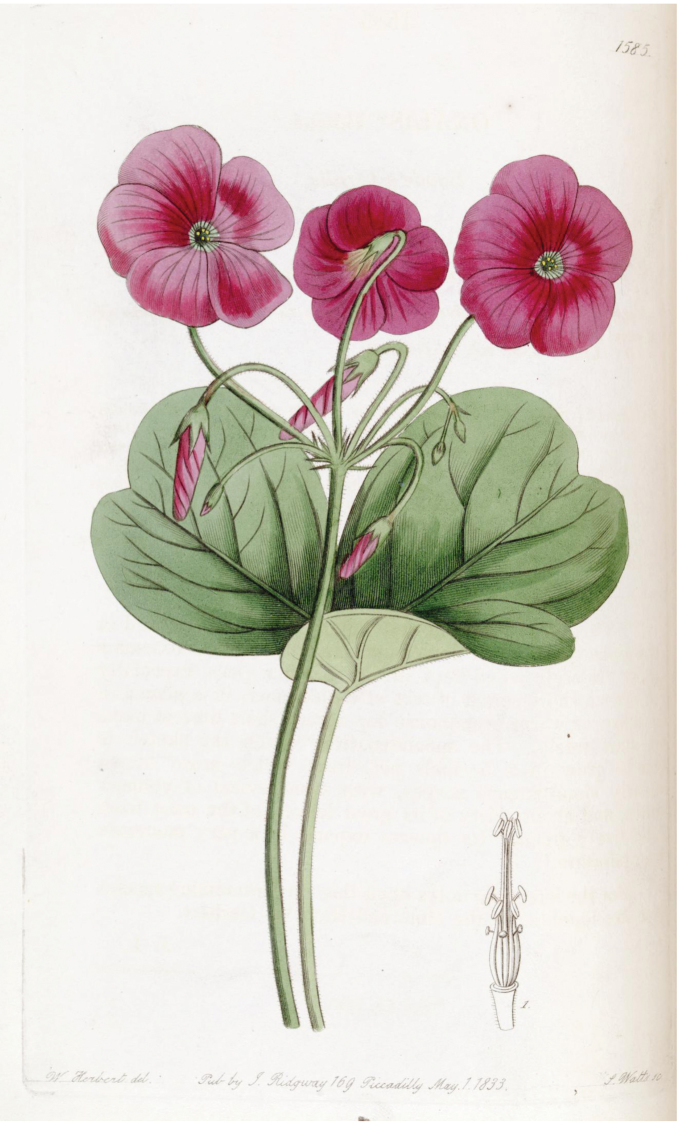
The illustration of *Oxalisbowiei* from Edward’s Botanical Register (Lindley 1833). https://biodiversitylibrary.org/page/239525

Occasionally the author citations *Oxalisbowiei* Herb. ex Lindl. or solely *Oxalisbowiei* Lindl. are used in the literature (Salter 1944). These are derived from Edward’s Botanical Register (Lindley 1833) (Fig. 2). However, this is a superfluous name under article 52 of the ICN, because it was published after Aiton’s name.

**Figure 3.**
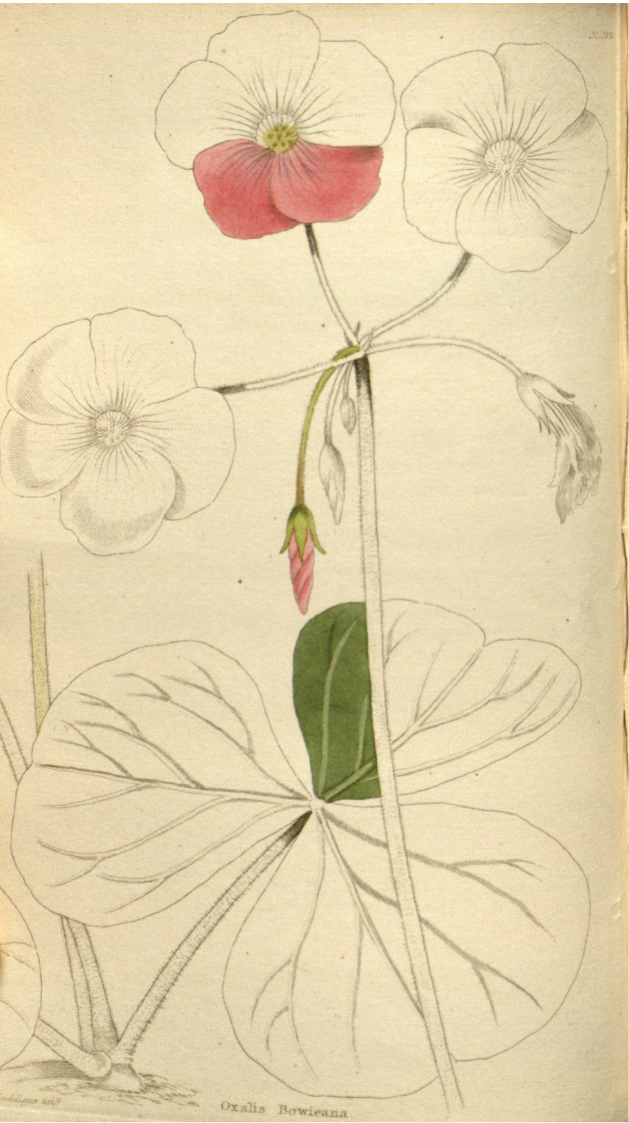
The illustration of *Oxalisbowiei* (as *Oxalisbowieana*) from The Botanical Cabinet (Loddiges 1832). https://biodiversitylibrary.org/page/29151292

Likewise, Loddiges (1832) (Fig. 3) uses the name *Oxalisbowieana*, either correcting Aiton’s name to the adjectival form or perhaps meaning to establish a new name, but again it was published after Aiton’s name (Don 1831). It is clear from the illustrations, names and descriptions that these versions of the name refer to the same taxon. Neither Lindley (1833) nor Loddiges (1832) mention any specimens, nor do they cite W.T.Aiton or G.Don.

## Typification

No herbarium specimens are mentioned in the description and the only reference to material, is that plants were cultivated from 1824 (“Clt.”), presumably at the Royal Botanic Gardens, Kew, where W.T.Aiton was director (Britten 1885) (Fig. 1). Therefore, this name was published without a holotype. Furthermore, I have been unable to find a subsequent typification where you might expect one, for example in publications on South African *Oxalis*, such as Salter (1944). The lack of a type has also been noted by Nesom (2017).

**Figure 4.**
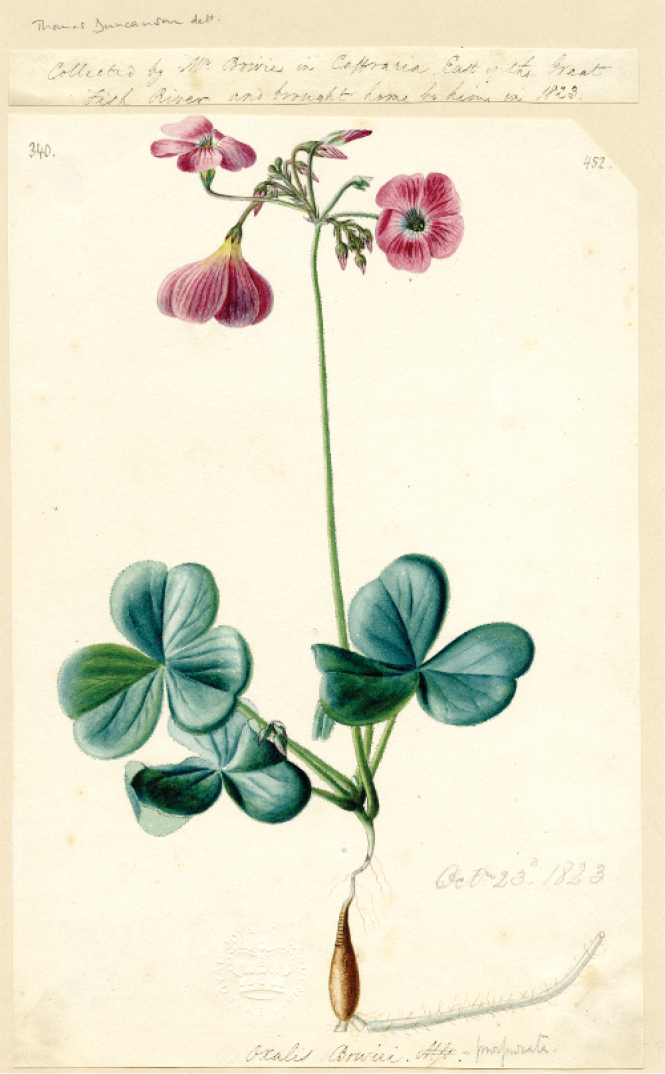
A painting of *Oxalisbowiei* by Thomas Duncanson deposited at the Royal Botanic Gardens, Kew. It is painted from material collected by James Bowie in the Eastern Cape, South Africa, East of the Great Fish River. Painted October 23^rd^ 1823. Number: 340, Alternative number: 452. Copyright The Trustees of the Royal Botanic Gardens, Kew.

There are no herbarium specimens at Kew for this species during this period. However, Thomas Duncanson was employed by W.T.Aiton at this time to paint plants newly imported and grown at Kew (Britten 1912). Indeed, there is a painting by Thomas Duncanson in the Kew Archives of *Oxalisbowiei* dated 23 October 1823, which may be a suitable type (Fig. 4). The requirements for lectotypification have been revised in the latest Code (McNeill et al. 2016, Turland et al. 2018). It is necessary to show that the illustration was “*available*” to the author and that the author “*associated*” the illustration with the taxon. Given the date and the location, it is clear that this comprises unpublished material that was available to W.T.Aiton prior to the preparation of the description. Also, as W.T.Aiton’s description mentions that the species was grown at Kew from 1824 and that the description matches the illustration, this satisfactorily associates the illustration with the description (Fig. 4).

A slight discrepancy is that the description states “*peduncles about equal in length to the leaves*”, whereas the Duncanson illustration shows a much longer peduncle. Nevertheless, Salter (1944) indicates that *O.bowiei* can have much longer peduncles and this trait certainly depends on cultural conditions. Otherwise, the Duncanson illustration is also the most accurate of the three illustrations reproduced here (Figs 2–4). It shows the underground structures, which are critical for correct identification. It also shows the absence of apical calli on the sepals (Salter 1944). Furthermore, the other figures imply that the sepals are fused over a third of their length, which is not the case.

Incidentally, other Duncanson illustrations have been used both as lectotypes and as neotypes for several other names of South African species (Figueiredo and Smith 2017; Glen and Hardy 2000; Smith 1990).

***Oxalisbowiei* W.T.Aiton ex G.Don, Gen. Hist. 1: 761, 1831 [early Aug 1831] (as “Bowii”)**

Lectotype, designated here: the illustration number 340 preserved at the Royal Botanic Gardens, Kew (K) (Alternative number: 452), painted by Thomas Duncanson, is designated as the lectotype of *Oxalisbowiei* W.T.Aiton ex G.Don (Fig. 4). This was from material collected by Bowie in Caffraria [Kaffraria], East of the Great Fish River and brought home by him in 1823. This illustration is of good quality, shows both above and below ground structures and has good detail and scale.
